# Effects of Oat Beta-Glucan Intake on Lipid Profiles in Hypercholesterolemic Adults: A Systematic Review and Meta-Analysis of Randomized Controlled Trials

**DOI:** 10.3390/nu14102043

**Published:** 2022-05-13

**Authors:** Junhui Yu, Jiayue Xia, Chao Yang, Da Pan, Dengfeng Xu, Guiju Sun, Hui Xia

**Affiliations:** Key Laboratory of Environmental Medicine and Engineering of Ministry of Education, Department of Nutrition and Food Hygiene, School of Public Health, Southeast University, Nanjing 210009, China; jshmyjh@126.com (J.Y.); 220203869@seu.edu.cn (J.X.); wenzhengwuguan@yeah.net (C.Y.); pantianqi92@foxmail.com (D.P.); withdrawxu@163.com (D.X.); gjunsun@seu.edu.cn (G.S.)

**Keywords:** oat beta-glucan, lipid profiles, hypercholesterolemia, meta-analysis

## Abstract

(1) Background: hyperlipidemia is one of the cardiovascular diseases which becomes a great threat to the health of people worldwide. Oat beta-glucan is reported to have a beneficial effect on lowering blood lipids. To probe the effect of oat beta-glucan consumption on serum lipid profiles (total cholesterol, total triglyceride, high-density lipoprotein-cholesterol, and low-density lipoprotein-cholesterol), we carried out a systematic search on randomized controlled trials of oat beta-glucan intervention on hypercholesterolemic individuals. (2) Methods: the pieces of literature were obtained from PubMed, Scopus, Cochrane Library, Web of Science, and the Embase from inception to 28 February 2022. The results were presented with the weighted mean difference (WMD) with a 95% CI. The random-effects or fixed-effects model was applied according to the heterogeneity. The subgroup analysis and meta-regression were used to identify the source of heterogeneity. (3) Results: thirteen trials with 927 participants were included in our meta-analysis. Overall, oat beta-glucan supplementation significantly reduced levels of TC (pooled WMD = −0.24 mmol/L; 95%CI: −0.28 to −0.20 mmol/L), LDL-c (pooled WMD = −0.27 mmol/L; 95%CI: −0.35 to −0.20 mmol/L). Furthermore, beta-glucan consumption did not show significant effects on TG (pooled WMD = −0.04 mmol/L; 95%CI: −0.13 to 0.05 mmol/L), HDL-c (pooled WMD = 0.00 mmol/L; 95%CI: −0.05 to 0.05 mmol/L). Subgroup analysis indicated that critical factors, such as disease severity of participants, the daily intervention of oat beta-glucan, source of oat beta-glucan, and duration of intervention had impacts on outcomes. (4) Conclusions: oat beta-glucan intake may significantly decrease the level of TC and LDL-c while no significant changes in TG and HDL-c were observed. This meta-analysis supports the health benefits of oat beta-glucan, especially for its cholesterol-lowering features, although it has some inevitable limitations.

## 1. Introduction

Cardiovascular diseases (CVD) have been an important cause of human death for decades. According to the report from the American Heart Association, the crude prevalence of CVD was 485.6 million cases in 2017, and this number increased by 28.5% over the past 10 years [1]. Cardiovascular diseases will continue to play a pivotal role in global mortality and the burden of disease. Although multiple factors can contribute to CVD, clinical and epidemiological studies demonstrate that dyslipidemia is a significant predictive factor for CVD, especially elevated low-density lipoprotein cholesterol (LDL-c), triglyceride levels, and lowered HDL-cholesterol (HDL-c) levels [2,3,4]. Although statins remain the preferred choice for patients with hypercholesterolemia, it does cause adverse effects, especially statin-associated muscle symptoms [5,6]. Thus, diet intervention was also recommended as a feasible method to improve dyslipidemia [7].

Oats, as one of the most cultivated and consumed whole grains worldwide, have been increasingly appreciated after adjustment to the dietary structure. With the progression of nutrition, oat was recognized as a healthy food in the mid-1980s signifying that a substance in it helped prevent heart disease and therefore it became popular for human nutrition [8]. Abundant secondary metabolites are synthesized in oat during ripening including beta-glucan. Beta-glucan, a viscous, soluble fiber, is distributed mainly in the endospermic cell wall of oats. It also widely exists in microalgae, fungi, and plants, and its efficacy tends to differ for minor changes in structures and junctional components. For example, β-glucans with β-(1→3) backbone and (1→6) linkages have immunostimulatory and anti-tumor properties [9]. As for cereal β-glucans, they are linked by a combination of β-(1→3) and β-(1→4) linkages [10,11]. Similarly, the immunostimulatory properties also presented in animal experiments. Oat β-glucan intervention significantly alleviated the symptoms of ulcerative colitis and inhibited mRNA and protein expression of several pro-inflammatory factors in mice [12]. In addition, oral administration of oat β-glucan in rats played a protective role against LPS-induced enteritis [13]. Furthermore, β-glucans linked via β-(1→3) were reported to help modulate glucose and cholesterol [14]. Authoritative bodies and professional societies such as the U.S. Food and Drug Administration (FDA), the Joint Health Claims Initiative (JHCI), and the French Food Safety Agency (AFFSA) already passed the health claims for oat beta-glucan.

Although Zhu et al. conducted a quantitative assessment to evaluate the effects of beta-glucan on lipid profiles in hypercholesterolemic subjects in 2015 [15], we found that several previous trials involved in this assessment had inappropriate controls and interventions which may have led to changes in lipid profiles in hypercholesterolemic patients. Hence, it is necessary to conduct a systematic review and meta-analysis of randomized controlled trials aiming to evaluate the effects of oat beta-glucan intake on lipid profiles in hypercholesterolemic adults according to all eligible studies.

## 2. Materials and Methods

### 2.1. Literature Search Strategy

We conducted our search according to The Preferred Reporting Items for Systematic Reviews and Meta-Analysis guidelines [16]. The search was carried out in PubMed, Web of Science, Embase, Scopus, and Cochrane library without language restrictions for relevant literature from inception to 28 February 2022. The terms we used were (“beta-Glucans” or ′′β-glucan” or “oat β-glucan” or “Glucans” or “′OBG” and “Hypercholesterolaemias” or “Hypercholesteremia” or “Dyslipidaemia” or “high cholesterol” or “high cholesterol level” or “elevated cholesterol”). For the limited relevant studies, we did not set any extra limitations in this strategy. We also conducted a hand-search on the reference lists in the potentially eligible literature to confirm any further eligible studies not retrieved in our prespecified database literature search. Two reviewers (J.Y. and J.X.) conducted the data extraction independently. Any disagreement on literature retrieval and literature inclusion was handled through district discussion and specialty consultation. The references to included studies were also investigated to ensure the rigor of the literature search strategy.

### 2.2. Study Selection

Studies were selected if they met the following inclusion criteria: (1) the design of study was a randomized controlled trial; (2) participants were patients aged over 18 years old with hypercholesterolemia and had no restrictions on complications; (3) trials using oat beta-glucan as an intervention to evaluate its effect on blood lipid profiles; (4) participants in the control group took a placebo or appropriate diet; (5) both the intervention group and control group had clear indicator values or numerical changes in blood lipid profiles.

The exclusion criteria were as follows: (1) objects involved in studies were animals or cells; (2) participants took drugs or other substances at the same time which might affect the level of the blood lipid profile; (3) trials without accurate data including total cholesterol (TC), total triglyceride (TG), high-density lipoprotein-cholesterol (HDL-c), and low-density lipoprotein-cholesterol (LDL-c) from baseline to endpoint; (4) blended interventions.

### 2.3. Data Extraction and Quality Assessment

Two reviewers (J.Y. and J.X.) extracted the following characteristics of 13 trials independently: first author, year, study region, study design, sample size (male/female), age, sources of β-glucan, amounts of β-glucan, duration, comparison group, baseline and endpoint of different lipid levels. The results of data extraction were aggregated after rigorous discussion.

Quality assessment of the studies included in this meta-analysis was performed according to the Cochrane collaboration risk-of-bias tool [17]. Seven domains are covered in this assessment tool (random sequence generation, allocation concealment, blinding of participants and personnel, blinding of outcome assessment, incomplete outcome data, selective outcome reporting, and other bias), and each item was scored as high, low, and unclear risk of bias based on the recommendations of the Cochrane Handbook.

### 2.4. Quantitative Data Synthesis and Analysis

We included trials with oat beta-glucan intervention or added oat beta-glucan to certain foods or the diet. Under the premise of meeting the previous inclusion and exclusion criteria, we split the trials into high and low doses and used “LD” to represent low doses and “HD” to represent high doses. For studies that did not provide direct data, we calculated the mean net change in blood lipid profiles by subtracting the mean change (end value minus baseline value). In addition, we calculated the mean difference (MD) in parallel-controlled trials by using the change from baseline in the control and intervention groups. If there were several endpoints in a study, we extracted the last value as the calculation. As for cross-over trials, the end of lipid profile levels in the control group and intervention group were selected to compute the MD in the effect. Data presented in milligrams per deciliter were transformed to millimoles per liter by dividing by 38.66 for HDL-c, LDL-c, and TC while 88.6 for TG. The formula was used if the report did not provide the net change of SD: SD_net change_ = $\sqrt{\mathrm{SD}{\left(\mathrm{baseline}\right)}^{2}+\mathrm{SD}{\left(\mathrm{endpoint}\right)}^{2}-2R\times \mathrm{SD}\left(\mathrm{baseline}\right)\times \;\mathrm{SD}\left(\mathrm{endpoint}\right)}$, based on a correlation coefficient R = 0.5 by Higgins et al. [17]. Meanwhile, for the literature that only reported median, sample size, and the first and the third quartiles, we adopted the following methods to estimate the mean and standard deviation: m (mean) = $\frac{q1+M+q3}{3}$, SD (standard deviation) = $\frac{q3-q1}{2*\Phi^{-1}\frac{0.75n-0.125}{n+0.25}}$ [7] (*q*^1^ for the 1st quartile, *M* for the median, *q*^3^ for the 3rd quartile), where *n* represents the sample size, and $\Phi^{-1}$ represents the inverse of the cumulative standard normal distribution). The test of statistical significance was two-sided: *p* < 0.05. Heterogeneity between trials was assessed by I^2^ and chi-squared statistics, either I^2^ > 50% or a *p*-value < 0.05 was considered as significant heterogeneity. In addition, a fixed-effects model was adopted to conduct the meta-analysis if statistical heterogeneity was present (*p* > 0.05 or I^2^ < 50%); otherwise, a random-effects model was used. Subgroup analyses were performed to explore the sources of heterogeneity. To identify which trial(s) led to the heterogeneity, a leave-one-out sensitivity analysis was adopted. In addition, we conducted subgroup analyses and regression based on the characteristics of disease severity, daily intervention dose, source of β-glucan, and duration. All statistical analyses above were performed using Stata version 15 (STATA Corp, College Station, TX, USA).

## 3. Results

### 3.1. Search Results and Characteristics of Included Trials

**Figure 1:**
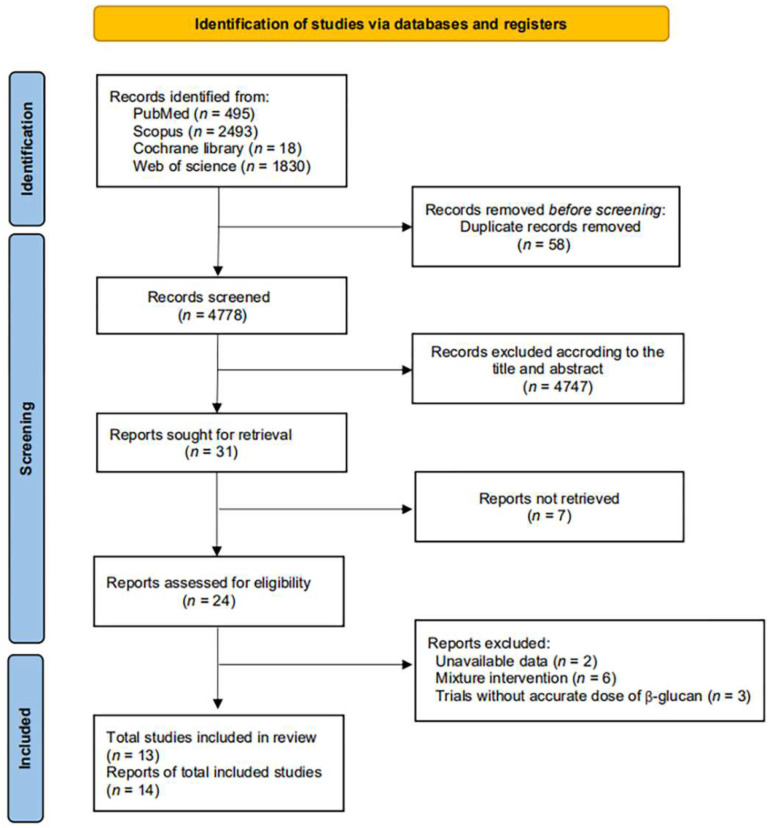
Flow chart.

Study selection and screening were documented and are presented in Figure 1; a total of 4836 articles were identified initially with the search terms from four databases, and 4747 of which were removed after reviewing the title and abstracts, including 58 duplications, leaving 31 trials for full manuscript review. After a detailed full-article review, 13 trials, spilt into 14 effect sizes, were included in the current systematic review and meta-analysis which met our inclusion criteria.

Overall, there are 13 RCTs summarized in Table 1 (Önning et al. 1999 [18]; Amundsen et al. 2003 [19]; Kerckhoffs et al., 2003 [20]; Martensson et al., 2005 [21]; KARMALLY et al., 2005 [22]; Reyna-Villasmil et al., 2007 [23]; Queenan et al., 2007 [24]; Biörklund, et al., 2008 [25]; Charlton et al., 2012 [26]; Zhang et al., 2012 [27]; Momenizadeh et al., 2014 [28]; Gulati et al., 2017 [29]; Xu et al., 2021 [30]). A total of 927 participants aged from 38 to 76 were randomized to oat beta-glucan or control groups. Most of the participants were mildly hypercholesterolemic individuals while the remainders were patients with varying degrees of hypercholesterolemia. Control groups received bread, rice, soup, and a diet without oat beta-glucan or oats as a placebo. The duration of intervention ranged from 3 weeks to 8 weeks with daily consumption of β-glucan varying from 1.5 g to 6 g. The majority of the trials were randomized, parallel and control design, and only one of these was a cross-over study. The baseline and endpoint of lipid profiles are also included in Table 1 and Table 2 for assessments of the risk of bias in studies involved.

### 3.2. Effect of Oat Beta-Glucan on Lipid Profiles

Thirteen studies investigated the effects of supplementing oat beta-glucan on TC, TG; ten studies probed the effects of oat beta-glucan consumption on HDL-c while twelve trials explored the effects of oat beta-glucan supplementation on LDL-c in patients with hypercholesterolemia.

Four trials reported that the intervention of oat beta-glucan had no significant effects on TC while ten remaining trials indicated that oat beta-glucan could lower the level of total cholesterol. However, when all the studies above were involved in our meta-analysis, the result revealed that consumption of oat beta-glucan did cause a significant reduction in TC in patients with hypercholesterolemia (mean difference = −0.24; 95% CI: −0.28 to −0.20; *p* = 0.000), and there exists no significant heterogeneity between all the trials (I^2^ = 38.9%; *p* = 0.068).

Two studies found that oat beta-glucan significantly decreased TG while two other trials reported that oat beta-glucan increased total triglyceride level. However, when all the data from included studies were pooled for meta-analysis, it revealed that consuming oat beta-glucan had no significant reduction in TG in patients with hypercholesterolemia (mean difference = −0.04; 95% CI: −0.13 to 0.05; *p* = 0.413). In addition, significant heterogeneity was exhibited between included trials (I^2^ = 90.3%; *p* = 0.000).

Reyna-Villasmil et al. reported that oat beta-glucan could remarkably increase HDL-c while Queenan et al. and Kerckhoffs et al. found that the oat beta-glucan group significantly decreased the level of HDL-c compared with the control. Moreover, when all the data from trials were included in the analysis, the results showed that oat beta-glucan intake had no significant effects on HDL-c in patients with hypercholesterolemia (mean difference = 0.00; 95% CI: −0.05 to 0.05; *p* = 0.993). In addition, heterogeneity between trials was significant (I^2^ = 97.7%; *p* = 0.000).

Eight included studies demonstrated that oat beta-glucan significantly decreased LDL-c while other trials did not. A cumulative meta-analysis of thirteen RCTs indicated a significant reduction in LDL-c after consuming oat beta-glucan in comparison with placebo-control groups in patients with hypercholesterolemia (mean difference = −0.27; 95% CI: −0.35 to −0.20; *p* = 0.000), and there was significant heterogeneity between the included trials (I^2^ = 83.3%; *p* = 0.000).

**Figure 2:**
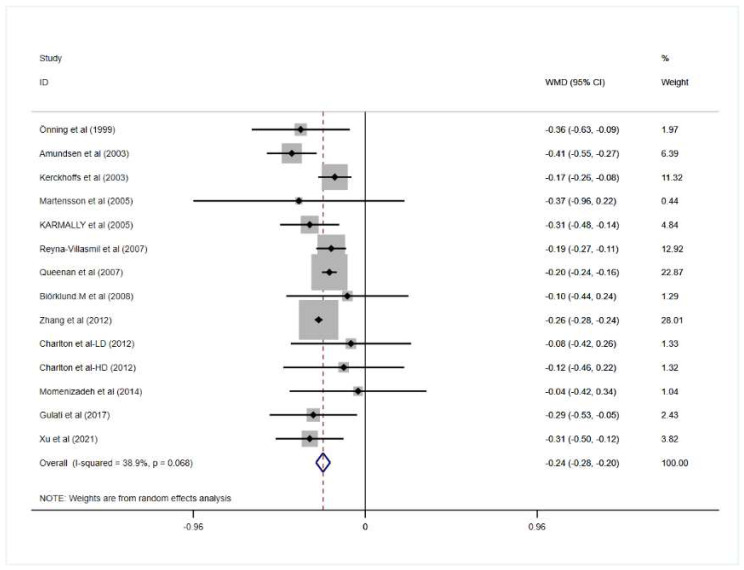
Effect of oat β-glucan on total cholesterol (mmol/L) in patients with hyperlipidemia. Open diamonds: synthesis of summary results of all studies; Filled diamonds: point estimates for effect of each study; Dashed lines: summary effect estimate which is labeled as a dashed line perpendicular to the X axis; Grey squares: the weight of each study, the value of weigh is proportional to the diamond size. WMD: weighted mean difference [18,19,20,21,22,23,24,25,26,27,28,29,30].

**Figure 3:**
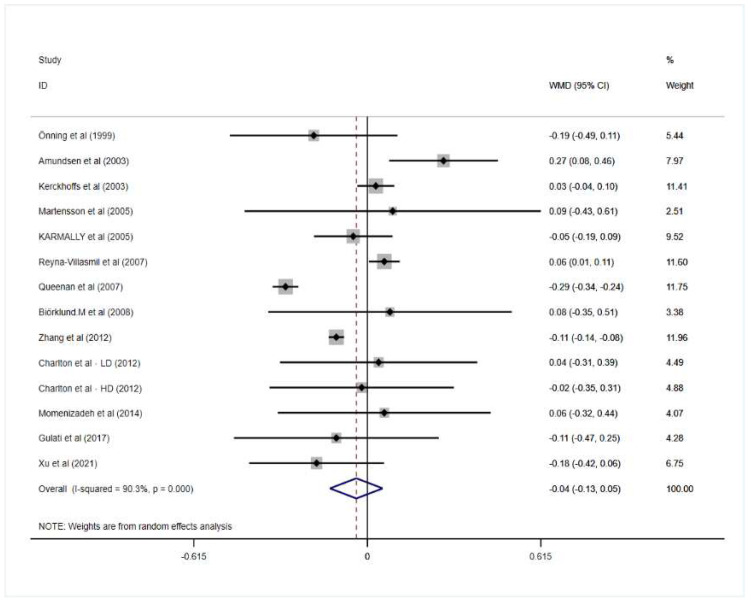
Effect of oat β-glucan on total triglyceride (mmol/L) in patients with hyperlipidemia. Open diamonds: synthesis of summary results of all studies; Filled diamonds: point estimates for effect of each study; Dashed lines: summary effect estimate which is labeled as a dashed line perpendicular to the X axis; Grey squares: the weight of each study, the value of weigh is proportional to the diamond size; WMD: weighted mean difference [18,19,20,21,22,23,24,25,26,27,28,29,30].

**Figure 4:**
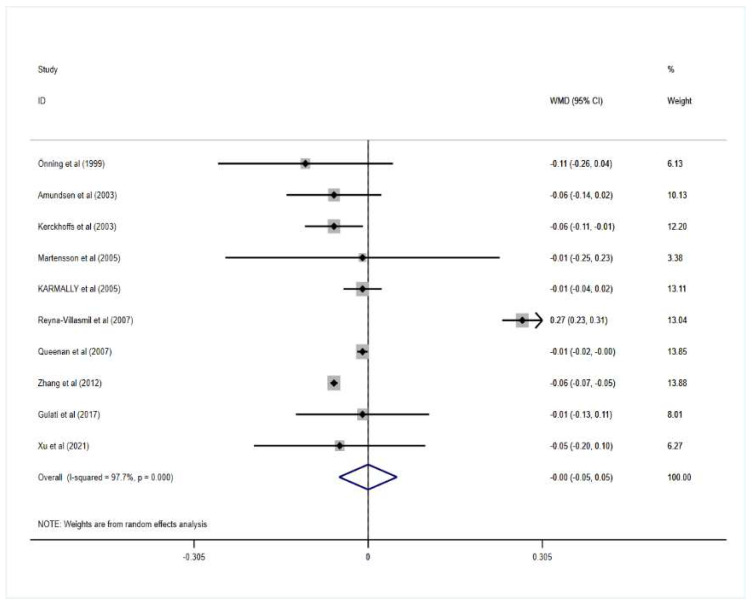
Effect of oat β-glucan on high-density lipoprotein-cholesterol (mmol/L) in patients with hyperlipidemia. Open diamonds: synthesis of summary results of all studies; Filled diamonds: point estimates for effect of each study; Grey squares: the weight of each study, the value of weigh is proportional to the diamond size; WMD: weighted mean difference. Arrow: effect size whose 95% confidence interval beyond the range of graphic display [18,19,20,21,22,23,24,25,26,27,28,29,30].

**Figure 5:**
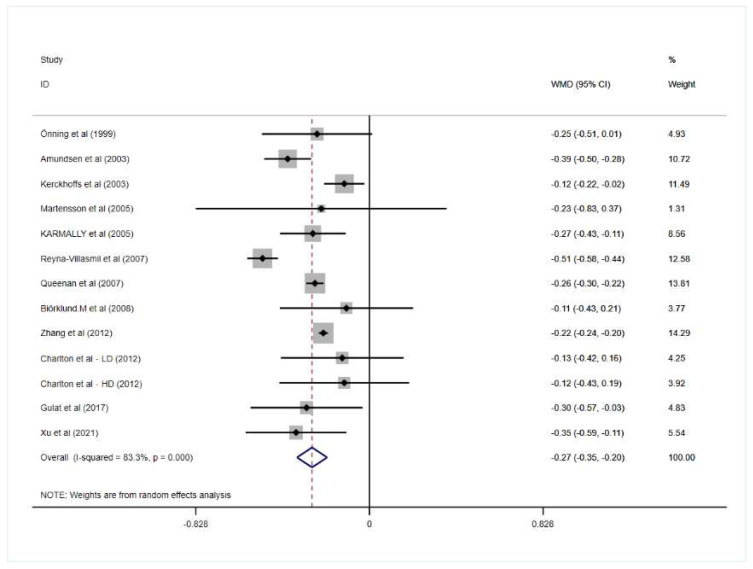
Effect of oat β-glucan on low-density lipoprotein-cholesterol (mmol/L) in patients with hyperlipidemia. Open diamonds: synthesis of summary results of all studies; Filled diamonds: point estimates for effect of each study; Dashed lines: summary effect estimate which is labeled as a dashed line perpendicular to the X axis; Grey squares: the weight of each study, the value of weigh is proportional to the diamond size; WMD: weighted mean difference [18,19,20,21,22,23,24,25,26,27,28,29,30].

More detailed results for the effect of oat beta-glucan on lipid profiles are presented in Figure 2, Figure 3, Figure 4 and Figure 5.

### 3.3. Subgroup Analysis and Meta-Regression 

The results of subgroup analysis are presented in Table 3. Oat beta-glucan intake could significantly reduce TG levels in subjects with moderate hypercholesterolemia while it did not induce great changes in mildly hypercholesterolemic patients (TG of mild patients: WMD = −0.01 mmol/L; 95% CI: −0.15 to 0.12 mmol/L, TG of moderate patients: WMD = −0.11 mmol/L; 95% CI: −0.14 to −0.08 mmol/L). In addition, the effects of oat beta-glucan on HDL-c tended to differ in two categories of patients since the level of HDL-c in moderately hypercholesterolemic subjects decreased more than another class of patients (HDL-c of mild patients: WMD = 0.01 mmol/L; 95% CI: −0.08 to 0.10 mmol/L, HDL-c of moderate patients: WMD = −0.06 mmol/L; 95% CI: −0.07 to −0.05 mmol/L). However, the differences in TC and LDL-c were subtle between them (TC of mild hypercholesterolemia patients: WMD = −0.22 mmol/L; 95% CI: −0.27 to −0.17 mmol/L, TC of moderate hypercholesterolemia patients: WMD = −0.26 mmol/L; 95% CI: −0.28 to −0.24 mmol/L; LDL-c of mild hypercholesterolemia patients: WMD = −0.28 mmol/L; 95%CI: −0.38 to −0.18 mmol/L, LDL-c of moderate hypercholesterolemia patients: WMD = −0.22 mmol/L; 95% CI: −0.24 to −0.20 mmol/L). Besides, different doses of oat beta-glucan had different effects on lipid profiles, especially TG (TG of daily intervention dose ≤3 g: WMD = −0.11 mmol/L; 95% CI: −0.13 to −0.08 mmol/L; TG of daily intervention dose < 3 g: WMD = 0.00 mmol/L; 95% CI: −0.16 to 0.16 mmol/L).

### 3.4. Publication Bias

**Figure 6:**
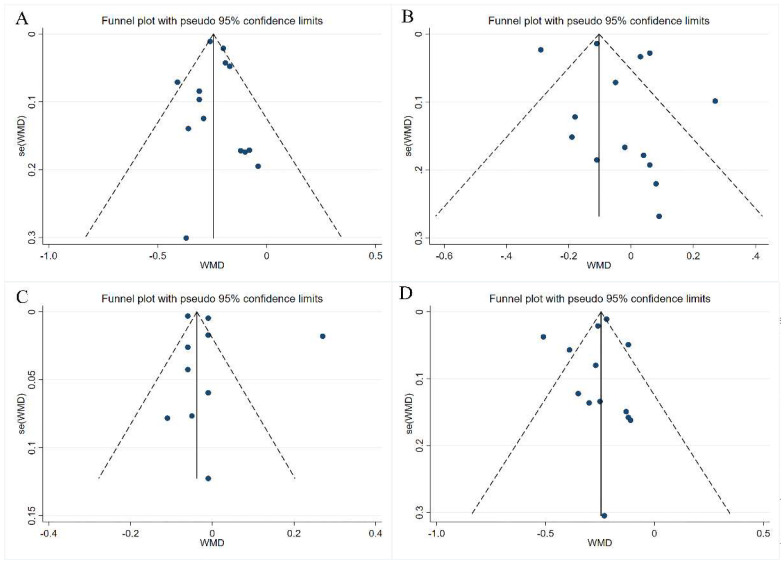
Funnel plots for exhibition of publication bias and oat β-glucan consumption for (**A**) TC Egger′s test (*p* = 0.637), (**B**) TG Egger′s test (*p* = 0.374), (**C**) HDL-C Egger’s test (*p* = 0.366), (**D**) LDL-C Egger′s test (*p* = 0.519). Solid line: combined effect size; Dashed line: 95% confidence interval.

Potential publication bias was measured by funnel plots and Egger’s regression test. We evaluated the symmetry of the funnel plots intuitively. Besides, a method of “trim and fill” analysis was conducted to evaluate publication bias further if there existed any asymmetry in the funnel plot [31]. Although there is a slight visual asymmetry in the funnel plot for TG, Egger’s regression *p*-value of 0.374 suggested that the results of TG are reliable. As for the other three indicators, visual scanning of funnel plots and Egger’s regression *p*-value of 0.637, 0.366, and 0.519 indicated an absence of publication bias, respectively. The funnel plots are depicted in Figure 6.

## 4. Discussion

It is well known that an elevated lipid profile is associated with the pathogenesis of cardiovascular diseases. Consuming more than 3 g oat beta-glucan each day is recommended by the FDA to gain its beneficial effects. This meta-analysis of 13 RCTs appraising the oat beta-glucan intake on lipid profiles indicated that oat beta-glucan intake has significant impacts on TC and LDL-c but not on TG and HDL-c. Despite the health benefits of consuming oat beta-glucan, few meta-analyses were found to evaluate its single effect on hypercholesterolemic individuals. Xu et al. conducted a meta-analysis focusing on the different effects of several delivering matrices of oat beta-glucan in mildly hypercholesterolemic subjects which concluded that a combination of “liquid” and “solid” products exerted the most remarkable effect [32]. Another recent meta-analysis in 2021 concluded that oats intake could reduce the risk of type 2 diabetes and all-cause mortality [33]. During our early retrieval and preliminary screening of the literature, we found that oat beta-glucan-based products were adopted in considerable trials. However, most of them were mixed interventions after our full-text screening, and we distinguished the single effect of oat beta-glucan on hypercholesterolemic patients from a certain number of pieces of the literature. Finally, thirteen eligible studies were included. The effects of oat beta-glucan on lipid profiles were similar in the present study compared with previous studies [15,32].

Based on the current subgroup analyses, we found several factors that were worth further exploration. As detailed above, only two studies targeting moderate hypercholesterolemic subjects were considered for inclusion. According to the degree of disease, we found the different effects of oat beta-glucan on lipid profiles. When we conducted a sensitivity analysis, we found that four major indicators of lipid profiles almost decreased significantly in these two trials compared with control groups while other trials targeting mild hypercholesterolemic subjects had controversial results [18,27]. This may suggest that oat beta-glucan intake played a more prominent role in improving lipid profiles in moderately hypercholesterolemic patients. Considering there was not enough relevant literature to support this view, therefore, we expect more trials focusing on this point. Furthermore, Queenan et al. found that oat beta-glucan consumption could significantly ameliorate the lipids level of females compared with males [24]. However, few studies reported the different effects of oat beta-glucan on men and women. A large, prospective, epidemiologic study formed the recommendations for fiber intake (25 and 38 g daily for young women and men). This study reported the different effects of fiber intake on men and women, suggesting gender differences [34]. Since oat beta-glucan belongs to dietary fiber, we had reasonable speculation: oat beta-glucan consumption had different effects on hypercholesterolemic men and women. Moreover, subgroup analysis on HDL-c showed the opposite effects between trials >5 weeks and ≤5 weeks. Given the small size of included trials and the longest trial only lasting 8 weeks, we were unclear about the potential explanations that account for this. In addition, significant heterogeneity still existed in some subgroups; we failed to explain them completely. The results of lipid profiles were robust in the sensitivity analysis.

Unlike general intervention, the effects of β-glucan could also be explained from a microscopic standpoint. Kerckhoffs et al. held the opinion that the effect was decreased owing to the bile acid reabsorption and different molecular weight β-glucans [20]. The consumption of β-glucan increased bile acid excretion which stimulates cholesterol metabolism and elimination. In addition, the measurement of cholesterol synthesis markers in blood suggests higher cholesterol elimination and lower absorption [35]. Compared with the intervention dose, the effects of oat beta-glucan on lipid profiles seem to deserve more attention on molecular weight (Mw). Smith et al. also conducted an intervention aiming to explore the different effects of high molecular weight and low molecular weight concentrated barley β-glucan. The results showed significant differences in the cholesterol/HDL ratio between the two groups; neither group showed benefits on lipid profiles [36]. However, high molecular weight oat β-glucan tends to have a higher viscosity in comparison with low molecular weight oat β-glucan which may therefore lead to great reductions in lipid profiles. Besides, the effect of oat beta-glucan in oat bran might decrease when compared with juice added with oat beta-glucan [20]. Hence, we considered this factor and divided all trials into two groups according to the source of oat β-glucan. To our surprise, we found that food or diet added with oat β-glucan may have a stronger effect on TC and TG than oat β-glucan from oats and oat-based products, although changes in TG were not statistically significant. In addition, the conclusion was opposite on LDL-c. 

Extrusion and processing probably improve the bioactivity of oat β-glucan [37]. Oat β-glucan from fermented, ropy, oat-based products significantly reduced the level of total cholesterol which indicated that food processing may also play a non-negligible role in the effect of oat β-glucan which resulted in different health impacts [21]. Likewise, Wang et al. believed that species, varieties, growing, storage conditions, and extraction might affect the structure and properties of oat β-glucan [38]. Furthermore, the amount, molecular weight, and solubility of oat β-glucan should also be considered when evaluating the effect of oat β-glucan on lipid profiles in hypercholesterolemic adults. Their attempts provided us with some new enlightenment for future explorations on oat β-glucan.

Beyond the effects of oat beta-glucan intake on lipid profiles in hypercholesterolemic adults, it was reported that oat β-glucan mixed with various vitamins and minerals in oat noodles could significantly reduce the level of both TC and LDL-c. In addition, a great decrease in many CVDs ratio markers was also witnessed in this trial [39]. Moreover, consuming oat β-glucan and plant stanol esters simultaneously reduced the LDL-c compared with the control group [40]. Several similar trials also indicated the lipid-lowering features of oat β-glucan and other substances. This may suggest the synergy of oat β-glucan and other nutrients and their health benefits. Last but not least, whether different sources of β-glucan have different biological functions or not remains to be mined.

There are several strengths in our meta-analysis. First, an examination of β-glucan′s single effect made it more powerful than any mixed intervention trial. Second, we comprehensively assessed the effect of oat β-glucan on lipid profiles in hypercholesterolemic adults; this provided a systematic view of elucidating its bioactive activity mechanism. Meanwhile, this meta-analysis focused on the gender differences and different severity levels of disease for the first time. We hope these findings based on current evidence will be a helpful contribution to a deeper exploration of oat β-glucan.

Simultaneously, the present review has several apparent limitations. First, we failed to find the precise sources of heterogeneity in TG, HDL-c, and LDL-c via subgroup analysis and meta-regression. This might be caused by other factors that we ignored. Second, owing to the careful screening of the including studies, trials using oats or oat bran that did not report a clear amount of oat β-glucan were omitted. The results of these trials may potentially affect our conclusion. Thirdly, this meta-analysis was not persuasive enough given its limited sample size.

## 5. Conclusions

Overall, the results from our analysis demonstrated that dietary consumption of oat beta-glucan may significantly reduce the level of TC and LDL-c in patients with hypercholesterolemia. Meanwhile, there is insufficient evidence to determine whether or not an association exists between oat beta-glucan intake and changes in TG and HDL-c levels. Therefore, we recommend that hypercholesterolemia patients take in adequate oat beta-glucan to help manage lipid profiles. Besides, more large-scale, high-quality randomized controlled trials are needed to support these findings since the current trials had inconsistent results.

## Figures and Tables

**Table 1:** Characteristics of included studies.

Number	First Author	Year	Study Region	Sample Size (Male/Female)	Age (Year)	Sources of Oatβ-Glucan	Amounts of Oatβ-Glucan (g/d)	Duration (Week)	Comparison Group	Study Design	Baseline of TC (mmol/L) (CG)	Baseline of TG (mmol/L) (CG)	Baseline of HDL-C (mmol/L) (CG)	Baseline of LDL-C (mmol/L) (CG)
1	Gunilla Önning	1999	Sweden	52 (52/0)	62.6 ± 5.6	oat milk	3.9	5	milk without oatβ-glucan	randomized controlled, double-blind	6.39 ± 0.68	1.56 ± 0.69	1.34 ± 0.33	4.34 ± 0.66
2	Ågot Lia Amundsen	2003	Sweden	16 (9/7)	57 ± 7.9	diet with oatβ-glucan	5	3	diet without oatβ-glucan	single-blind, randomized, cross-over	7.64 ± 0.24	2.15 ± 0.37	1.68 ± 0.13	5.12 ± 0.20
3	Daniëlle AJM Kerckhoffs	2003	Netherland	48 (21/27)	51.3 ± 2	bread and cookies with oatβ-glucan	5	4	bread and cookies without oatβ-glucan	parallel, randomized controlled	6.00 ± 0.16	1.15 ± 0.09	1.38 ± 0.09	4.09 ± 0.17
4	Olof Martensson	2005	Sweden	56 (24/32)	55.0 ± 9.0	Oat-based product	3.25	5	dairy-based product	randomized, double blind, parallel	6.04 ± 1.02	1.51 ± 0.44	1.21 ± 0.32	4.09 ± 1.19
5	WAHIDA KARMALLY	2005	US	152 (49/103)	49.1 ± 10.3	oat-containing cereal	3	6	corn	randomized, controlled trial	5.15 ± 0.72	1.64 ± 0.73	0.96 ± 0.23	3.45 ± 0.63
6	Nadia Reyna-Villasmil	2007	Venezuela	38 (38/0)	59.8 ± 0.6	oat bread	6	8	whole wheat bread	randomized, controlled trial	6.02 ± 0.07	1.44 ± 0.11	1.09 ± 0.07	4.15 ± 0.07
7	Katie M Queenan	2007	US	75 (25/50)	45.0 ± 2.1	concentrated oatβ-glucan	6	6	dextrose monohydrate	randomized, double-blind parallel	6.20 ± 0.10	1.90 ± 0.20	1.30 ± 0.05	4.20 ± 0.10
8	M. Biörklund	2008	Sweden	43 (19/24)	58.0 ± 8.2	soup with oatβ-glucan	4	5	soup without oatβ-glucan	parallel, placebo-controlled	6.66 ± 0.52	1.54 ± 0.66	NR	4.06 ± 0.52
9	Karen E. Charlton	2012	Australia	90 (43/47)	51.3 ± 10.22	oat porridge, oat-based cereal bars	1.5 (LD), 3.2 (HD)	6	diet without oatβ-glucan	parallel, randomized, controlled, single-blind	6.03 ± 0.58	1.56 ± 0.60	NR	3.86 ± 0.55
10	Jian Zhang	2012	China	166 (65/101)	53.19 ± 0.87	diet with oatβ-glucan	3	6	diet without oatβ-glucan	randomized, controlled	6.09 ± 0.08	1.89 ± 0.11	1.51 ± 0.03	4.17 ± 0.08
11	Amir Momenizadeh	2014	Iran	60 (21/39)	51.12 ± 9.31	oat bread	6	6	wheat bread oatβ-glucan	randomized, controlled	5.80 ± 0.45	1.95 ± 0.67	NR	NR
12	Seema Gulati	2017	US	69	31.2 ± 6.54	porridge and upma with oatβ-glucan	3	4	diet without oatβ-glucan	prospective, randomized, parallel, controlled	5.62 ± 0.29	1.78 ± 0.63	1.01 ± 0.21	3.76 ± 0.46
13	Dengfeng Xu	2021	China	62 (22/40)	44.69 ± 11.42	oats	3	6	rice without oatβ-glucan	randomized, controlled	5.55 ± 0.29	1.22 ± 0.43	1.53 ± 0.30	3.30 ± 0.41
**Number**	**First** **Author**	**Year**	**Endpoint of TC** **(mmol/L) (CG)**	**Endpoint of TG** **(mmol/L) (CG)**	**Endpoint of HDL-C** **(mmol/L) (CG)**	**Endpoint of LDL-C** **(mmol/L) (CG)**	**Baseline of TC** **(mmol/L) (IG)**	**Baseline of TG** **(mmol/L) (IG)**	**Baseline of HDL-C** **(mmol/L) (IG)**	**Baseline of LDL-C** **(mmol/L) (IG)**	**Endpoint of TC(mmol/L)(IG)**	**Endpoint of TG** **(mmol/L) (IG)**	**Endpoint of HDL-C** **(mmol/L) (IG)**	**Endpoint of LDL-C** **(mmol/L) (IG)**
1	Gunilla Önning	1999	6.58 ± 0.80	1.85 ± 0.92	1.39 ± 0.34	4.38 ± 0.82	6.42 ± 0.66	1.57 ± 0.74	1.43 ± 0.51	4.35 ± 0.65	6.25 ± 0.67	1.67 ± 0.67	1.37 ± 0.33	4.14 ± 0.56
2	Ågot Lia Amundsen	2003	7.34 ± 0.18	1.68 ± 0.21	1.53 ± 0.11	5.11 ± 0.15	7.66 ± 0.19	1.77 ± 0.25	1.74 ± 0.13	5.19 ± 0.14	6.95 ± 0.16	1.57 ± 0.20	1.53 ± 0.11	4.79 ± 0.14
3	Daniëlle AJM Kerckhoffs	2003	6.04 ± 0.15	1.12 ± 0.10	1.41 ± 0.09	4.11 ± 0.16	5.98 ± 0.16	1.13 ± 0.12	1.50 ± 0.09	3.96 ± 0.16	5.85 ± 0.18	1.13 ± 0.14	1.47 ± 0.08	3.86 ± 0.17
4	Olof Martensson	2005	6.15 ± 1.00	1.46 ± 0.52	1.29 ± 0.32	4.11 ± 1.12	6.08 ± 0.75	1.52 ± 1.09	1.36 ± 0.41	3.78 ± 0.73	5.82 ± 0.80	1.56 ± 0.96	1.43 ± 0.40	3.57 ± 0.80
5	WAHIDA KARMALLY	2005	5.18 ± 0.70	1.63 ± 0.75	0.97 ± 0.23	3.48 ± 0.58	5.41 ± 0.77	1.79 ± 0.73	0.93 ± 0.25	3.66 ± 0.68	5.12 ± 0.65	1.71 ± 0.71	0.93 ± 0.26	3.41 ± 0.56
6	Nadia Reyna-Villasmil	2007	5.24 ± 0.17	1.35 ± 0.08	1.08 ± 0.06	3.44 ± 0.14	6.00 ± 0.11	1.27 ± 0.05	1.02 ± 0.05	4.34 ± 0.11	5.02 ± 0.11	1.24 ± 0.08	1.28 ± 0.05	3.12 ± 0.11
7	Katie M Queenan	2007	6.10 ± 0.10	1.70 ± 0.15	1.29 ± 0.05	4.2 ± 0.07	6.20 ± 0.10	1.90 ± 0.10	1.40 ± 0.06	4.10 ± 0.10	5.90 ± 0.10	1.99 ± 0.10	1.38 ± 0.06	3.80 ± 0.10
8	M. Biörklund	2008	6.57 ± 0.60	1.51 ± 0.64	NR	4.01 ± 0.60	6.62 ± 0.51	1.58 ± 0.81	NR	4.29 ± 0.50	6.43 ± 0.63	1.63 ± 0.76	NR	4.13 ± 0.50
9	Karen E. Charlton	2012	5.67 ± 0.68	1.55 ± 0.78	NR	3.60 ± 0.53	6.12 ± 0.54 (LD) 5.97 ± 0.55 (HD)	1.53 ± 0.73 (LD) 1.37 ± 0.59 (HD)	NR	3.84 ± 0.67 (LD) 3.82 ± 0.56 (HD)	5.68 ± 0.77 (LD) 5.49 ± 0.80 (HD)	1.56 ± 0.58 (LD) 1.34 ± 0.60 (HD)	NR	3.49 ± 0.70 (LD) 3.46 ± 0.69 (HD)
10	Jian Zhang	2012	5.94 ± 0.09	1.85 ± 0.11	1.41 ± 0.03	4.00 ± 0.08	6.26 ± 0.07	2.06 ± 0.10	1.47 ± 0.03	1.47 ± 0.03	5.85 ± 0.09	1.91 ± 0.11	1.43 ± 0.03	3.91 ± 0.08
11	Amir Momenizadeh	2014	5.67 ± 0.88	1.87 ± 0.84	NR	NR	6.08 ± 0.70	2.03 ± 0.71	NR	NR	5.89 ± 0.80	2.00 ± 0.72	NR	NR
12	Seema Gulati	2017	5.40 ± 0.63	1.92 ± 0.91	0.96 ± 0.19	3.56 ± 0.74	5.62 ± 0.28	1.83 ± 0.67	1.08 ± 0.33	3.73 ± 0.41	5.11 ± 0.56	1.85 ± 0.76	1.02 ± 0.25	3.23 ± 0.52
13	Dengfeng Xu	2021	5.39 ± 0.46	1.24 ± 0.51	1.56 ± 0.35	3.20 ± 0.59	5.59 ± 0.30	1.34 ± 0.50	1.49 0.28	3.48 ± 0.44	5.12 ± 0.40	1.18 ± 0.45	1.47 ± 0.26	3.03 ± 0.45

Annotation: NR: not report; LD: low dose; HD: high dose; TC: total cholesterol; TG: total triglyceride; HDL-c: high-density lipoprotein-cholesterol; LDL-c: low-density lipoprotein-cholesterol; CG: control group; IG: intervention group.

**Table 2:** Risk of bias assessment.

Study	Year	Risk of Bias Assessment						
		Random Sequence Generation	Allocation Concealment	Blinding of Participants and Personnel	Blinding of Outcome Assessment	Incomplete Outcome Data	Selective Reporting	Other Bias
Önning et al.	1999	High	Low	Low	Low	Low	Low	Low
Amundsen et al.	2003	Unclear	Unclear	Low	Low	Low	Low	Low
Kerckhoffs et al.	2003	High	Low	High	Low	Low	Low	Unclear
Martensson et al.	2005	Unclear	Low	Low	Low	Low	Low	Unclear
Karmally et al.	2005	Unclear	Low	Low	Low	Low	Low	Unclear
Reyna-Villasmil et al.	2007	Unclear	Low	Low	Low	Low	Low	Low
Queenan et al.	2007	Low	Low	Low	Low	Low	Low	Low
Biörklund et al.	2008	Low	Low	Low	Low	Low	Low	Low
Zhang et al.	2012	Low	Low	Low	Low	Low	Low	Low
Charlton et al.	2012	Low	Low	Low	Low	Low	Low	Low
Momenizadeh et al.	2014	Low	Low	Low	Low	Low	Low	Low
Gulat et al.	2017	Unclear	High	High	Low	Low	Low	Unclear
Xu et al.	2021	Low	Low	Low	Low	Low	Low	Low

**Table 3:** Summary results of subgroup analyses.

Lipid Profiles	Subgroup Analyses					
	No. of Trials	WMD		*p*	I^2^ (%)	*p* Value of Heterogeneity
		Mean	95% CI			
TC						
Overall	14	−0.24	−0.28, −0.20	0.000	38.9	0.068
Disease severity						
mild	12	−0.22	−0.27, −0.17	0.000	22.8	0.219
moderate	2	−0.26	−0.28, −0.24	0.000	0.0	0.475
Daily intervention dose (g)						
≤3	5	−0.26	−0.28, −0.24	0.000	0.0	0.777
>3	9	−0.22	−0.27, −0.16	0.000	32.8	0.156
Source of oat β-glucan						
oats and oat-based products	8	−0.22	−0.29, −0.16	0.000	0.0	0.585
food or diet added with oat β-glucan	6	−0.24	−0.29, −0.19	0.000	66.9	0.010
Duration						
≤5 weeks	6	−0.28	−0.40, −0.16	0.000	47.5	0.090
>5 weeks	8	−0.23	−0.27, −0.19	0.000	40.3	0.110
TG						
Overall	14	−0.04	−0.13, 0.05	0.413	90.3	0.000
Disease severity						
mild	12	−0.01	−0.15, 0.12	0.846	91.7	0.000
moderate	2	−0.11	−0.14, −0.08	0.000	0.0	0.599
Daily intervention dose (g)						
≤3	5	−0.11	−0.13, −0.08	0.000	0.0	0.787
>3	9	0.00	−0.16, 0.16	0.983	93.9	0.000
Source of oat β-glucan						
oats and oat-based products	8	0.01	−0.05, 0.07	0.732	8.6	0.364
food or diet added with oat β-glucan	6	−0.05	−0.18, 0.09	0.498	94.5	0.000
Duration						
≤5 weeks	6	0.05	−0.08, 0.18	0.471	41.6	0.128
>5 weeks	8	−0.08	−0.20, 0.03	0.157	92.9	0.000
HDL-c						
Overall	10	0.00	−0.05, 0.05	0.993	97.7	0.000
Disease severity						
mild	8	0.01	−0.08, 0.10	0.784	97.1	0.000
moderate	2	−0.06	−0.07, −0.05	0.000	0.0	0.523
Daily intervention dose (g)						
≤3	4	−0.04	−0.08, 0.00	0.063	66.5	0.030
>3	6	0.01	−0.12, 0.14	0.890	97.9	0.000
Source of oat β-glucan						
oats and oat-based products	5	0.03	−0.15, 0.20	0.775	97.2	0.000
food or diet added with oat β-glucan	5	−0.04	−0.08, 0.00	0.029	95.1	0.000
Duration						
≤5 weeks	5	−0.06	−0.10, −0.02	0.004	0.0	0.870
>5 weeks	5	0.03	−0.03, 0.10	0.309	99.0	0.000
LDL-c						
Overall	13	−0.27	−0.35, −0.20	0.000	83.3	0.000
Disease severity						
mild	11	−0.28	−0.38, −0.18	0.000	82.0	0.000
moderate	2	−0.22	−0.24, −0.20	0.000	0.0	0.824
Daily intervention dose (g)						
≤3	5	−0.22	−0.24, −0.20	0.000	0.0	0.700
>3	8	−0.28	−0.40, −0.15	0.000	87.0	0.000
Source of oat β-glucan						
oats and oat-based products	7	−0.30	−0.44, −0.15	0.001	68.5	0.004
food or diet added with oat β-glucan	6	−0.24	−0.30, −0.18	0.000	69.8	0.005
Duration						
≤5 weeks	6	−0.24	−0.38, −0.10	0.001	63.5	0.018
>5 weeks	7	−0.29	−0.39, −0.20	0.000	89.7	0.000

Annotation: We split the trials with low and high doses and used “LD” to represent low doses and “HD” to represent high doses which resulted in 14 effect sizes in subgroup analyses. As for TG, HDL-c, and LDL-c, no significant reduction in heterogeneity was observed by subgroup analysis. We also found several trials that significantly affected the conclusions via sensitivity analyses. Therefore, we continued to conduct the meta-regression but did not find the possible sources of significant heterogeneity. TC: total cholesterol; TG: total triglyceride; HDL-c: high-density lipoprotein-cholesterol; LDL-c: low-density lipoprotein-cholesterol.

## Data Availability

Not applicable.

## References

[1] Virani S.S., Alonso A., Benjamin E.J., Bittencourt M.S., Callaway C.W., Carson A.P., Chamberlain A.M., Chang A.R., Cheng S., Delling F.N. (2020). Heart Disease and Stroke Statistics—2020 Update: A Report from the American Heart Association. Circulation.

[2] Ballantyne C., Arroll B., Shepherd J. (2005). Lipids and CVD management: Towards a global consensus. Eur. Heart J..

[3] Murray C.J.L., Lopez A.D. (1997). Alternative projections of mortality and disability by cause 1990–2020: Global Burden of Disease Study. Lancet.

[4] Yusuf S., Hawken S., Ôunpuu S., Dans T., Avezum A., Lanas F., McQueen M., Budaj A., Pais P., Varigos J. (2004). Effect of potentially modifiable risk factors associated with myocardial infarction in 52 countries (the INTERHEART study): Case-control study. Lancet.

[5] Bruckert E., Hayem G., Dejager S., Yau C., Begaud B. (2005). Mild to moderate muscular symptoms with high-dosage statin therapy in hyperlipidemic patients—The PRIMO study. Cardiovasc. Drugs Ther..

[6] Cohen J.D., Brinton E.A., Ito M.K., Jacobson T.A. (2012). Understanding Statin Use in America and Gaps in Patient Education (USAGE): An internet-based survey of 10,138 current and former statin users. J. Clin. Lipidol..

[7] Wan X., Wang W., Liu J., Tong T. (2014). Estimating the sample mean and standard deviation from the sample size, median, range and/or interquartile range. BMC Med. Res. Methodol..

[8] Sadiq Butt M., Tahir-Nadeem M., Khan M.K., Shabir R., Butt M.S. (2008). Oat: Unique among the cereals. Eur. J. Nutr..

[9] Du B., Lin C., Bian Z., Xu B. (2015). An insight into anti-inflammatory effects of fungal beta-glucans. Trends Food Sci. Technol..

[10] Izydorczyk M.S., Macri L.J., MacGregor L.W. (1998). Structure and physicochemical properties of barley non-starch polysaccharides—I. Water extractable β-Glucans and arabinoxylans. Carbohydr. Polym..

[11] Henry R.J. (1987). Pentosan and (1 → 3),(1 → 4)-β-Glucan Concentrations in Endosperm and Whole grain of Wheat, Barley, Oats and Rye. J. Cereal Sci..

[12] Liu B., Lin Q., Yang T., Zeng L., Shi L., Chen Y., Luo F. (2015). Oat beta-glucan ameliorates dextran sulfate sodium (DSS)-induced ulcerative colitis in mice. Food Funct..

[13] Błaszczyk K., Gajewska M., Wilczak J., Kamola D., Majewska A., Harasym J., Gromadzka-Ostrowska J. (2018). Oral administration of oat beta-glucan preparations of different molecular weight results in regulation of genes connected with immune response in peripheral blood of rats with LPS-induced enteritis. Eur. J. Nutr..

[14] Henrion M., Francey C., Le K.A., Lamothe L. (2019). Cereal B-Glucans: The Impact of Processing and How It Affects Physiological Responses. Nutrients.

[15] Zhu X., Sun X., Wang M., Zhang C., Cao Y., Mo G., Liang J., Zhu S. (2015). Quantitative assessment of the effects of beta-glucan consumption on serum lipid profile and glucose level in hypercholesterolemic subjects. Nutr. Metab. Cardiovasc. Dis..

[16] Moher D., Liberati A., Tetzlaff J., Altman D.G., The PRISMA Group (2009). Preferred Reporting Items for Systematic Reviews and Meta-Analyses: The PRISMA Statement. BMJ.

[17] Higgins J.P., Altman D.G., Gotzsche P.C., Juni P., Moher D., Oxman A.D., Savovic J., Schulz K.F., Weeks L., Sterne J.A. (2011). The Cochrane Collaboration’s tool for assessing risk of bias in randomised trials. BMJ.

[18] Onning G., Wallmark A., Persson M., Akesson B., Elmstahl S., Oste R. (1999). Consumption of oat milk for 5 weeks lowers serum cholesterol and LDL cholesterol in free-living men with moderate hypercholesterolemia. Ann. Nutr. Metab..

[19] Amundsen A.L., Haugum B., Andersson H. (2003). Changes in serum cholesterol and sterol metabolites after intake of products enriched with an oat bran concentrate within a controlled diet. Scand. J. Nutr..

[20] Kerckhoffs D.A.J.M., Hornstra G., Mensink R.P. (2003). Cholesterol-lowering effect of β-glucan from oat bran in mildly hypercholesterolemic subjects may decrease when β-glucan is incorporated into bread and cookies. Am. J. Clin. Nutr..

[21] Martensson O., Biorklund M., Lambo A.M., Duenas-Chasco M., Irastorza A., Holst O., Norin E., Welling G., Oste R., Onning G. (2005). Fermented, ropy, oat-based products reduce cholesterol levels and stimulate the bifidobacteria flora in humans. Nutr. Res..

[22] Karmally W., Montez M.G., Palmas W., Martinez W., Branstetter A., Ramakrishnan R., Holleran S.F., Haffner S.M., Ginsberg H.N. (2005). Cholesterol-lowering benefits of oat-containing cereal in Hispanic Americans. J. Am. Diet. Assoc..

[23] Reyna-Villasmil N., Bermudez V., Mengual-Moreno E., Arias N., Cano-Ponce C., Leal-Gonzalez E., Souki A., E Inglett G., Israili Z.H., Hernández-Hernández R. (2007). Oat-derived beta-glucan significantly improves HDLC and diminishes LDLC and non-HDL cholesterol in overweight individuals with mild hypercholesterolemia. Am. J. Ther..

[24] Queenan K.M., Stewart M.L., Smith K.N., Thomas W., Fulcher R.G., Slavin J.L. (2007). Concentrated oat β-glucan, a fermentable fiber, lowers serum cholesterol in hypercholesterolemic adults in a randomized controlled trial. Nutr. J..

[25] Biorklund M., Holm J., Onning G. (2008). Serum lipids and postprandial glucose and insulin levels in hyperlipidemic subjects after consumption of an oat beta-glucan-containing ready meal. Ann. Nutr. Metab..

[26] Charlton K.E., Tapsell L.C., Batterham M.J., O’Shea J., Thorne R., Beck E., Tosh S.M. (2012). Effect of 6 weeks’ consumption of β-glucan-rich oat products on cholesterol levels in mildly hypercholesterolaemic overweight adults. Br. J. Nutr..

[27] Zhang J., Li L., Song P., Wang C., Man Q., Meng L., Cai J., Kurilich A. (2012). Randomized controlled trial of oatmeal consumption versus noodle consumption on blood lipids of urban Chinese adults with hypercholesterolemia. Nutr. J..

[28] Momenizadeh A., Heidari R., Sadeghi M., Tabesh F., Ekramzadeh M., Haghighatian Z., Golshahi J., Baseri M. (2014). Effects of oat and wheat bread consumption on lipid profile, Blood sugar, And endothelial function in hypercholesterolemic patients: A randomized controlled clinical trial. ARYA Atheroscler..

[29] Gulati S., Misra A., Pandey R.M. (2017). Effects of 3 g of soluble fiber from oats on lipid levels of Asian Indians—A randomized controlled, parallel arm study. Lipids Health Dis..

[30] Xu D.F., Wang S.K., Feng M.Y., Shete V., Chu Y.F., Kamil A., Yang C., Liu H.C., Xia H., Wang X. (2021). Serum Metabolomics Reveals Underlying Mechanisms of Cholesterol-Lowering Effects of Oat Consumption: A Randomized Controlled Trial in a Mildly Hypercholesterolemic Population. Mol. Nutr. Food Res..

[31] Duval S. (1999). Trim and Fill: A Simple Funnel-Plot-Based Method of Testing and Adjusting for Publication Bias in Meta-Analysis. Biometrics.

[32] Xu D., Liu H., Yang C., Xia H., Pan D., Yang X., Yang L., Wang S., Sun G. (2021). Effects of different delivering matrices of beta-glucan on lipids in mildly hypercholesterolaemic individuals: A meta-analysis of randomised controlled trials. Br. J. Nutr..

[33] Wehrli F., Taneri P.E., Bano A., Bally L., Blekkenhorst L.C., Bussler W., Metzger B., Minder B., Glisic M., Muka T. (2021). Oat Intake and Risk of Type 2 Diabetes, Cardiovascular Disease and All-Cause Mortality: A Systematic Review and Meta-Analysis. Nutrients.

[34] Rebello C.J., Chu Y.F., Johnson W.D., Martin C.K., Han H., Bordenave N., Shi Y., O’Shea M., Greenway F.L. (2014). The role of meal viscosity and oat β-glucan characteristics in human appetite control: A randomized crossover trial. Nutr. J..

[35] Ellegard L., Andersson H. (2007). Oat bran rapidly increases bile acid excretion and bile acid synthesis: An ileostomy study. Eur. J. Clin. Nutr..

[36] Smith K.N., Queenan K.M., Fulcher R.G., Slavin J.L., Thomas W. (2008). Physiological Effects of Concentrated Barley β-Glucan in Mildly Hypercholesterolemic Adults. J. Am. Coll. Nutr..

[37] Tosh S.M., Brummer Y., Miller S.S., Regand A., Defelice C., Duss R., Wolever T.M., Wood P.J. (2010). Processing affects the physicochemical properties of beta-glucan in oat bran cereal. J. Agric. Food Chem..

[38] Wang Q., Ellis P.R. (2014). Oat beta-glucan: Physico-chemical characteristics in relation to its blood-glucose and cholesterol-lowering properties. Br. J. Nutr..

[39] Liao M.Y., Shen Y.C., Chiu H.F., Ten S.M., Lu Y.Y., Han Y.C., Venkatakrishnan K., Yang S.F., Wang C.K. (2019). Down-regulation of partial substitution for staple food by oat noodles on blood lipid levels: A randomized, double-blind, clinical trial. J. Food Drug Anal..

[40] Theuwissen E., Mensink R.P. (2006). Simultaneous Intake of b-Glucan and Plant Stanol Esters Affects Lipid Metabolism in Slightly Hy percholesterolemic Subjects. J. Nutr..

